# An “arboreal” infective pseudoaneurysm following TAVR with “pseudovascular” distribution and morphology

**DOI:** 10.1007/s12574-022-00571-y

**Published:** 2022-04-06

**Authors:** Kimberly R. Ding, Rod Partow, Narut Prasitlumkum, Padmini Varadarajan, Ramdas G. Pai

**Affiliations:** 1 School of Medicine, UC Riverside, University of California, Riverside, SOM Education Building, #2619, 900 University Avenue, Riverside, CA USA; 2 Department of Cardiology, University of California Riverside, Riverside, CA USA

## Case report

**Fig. 1:**
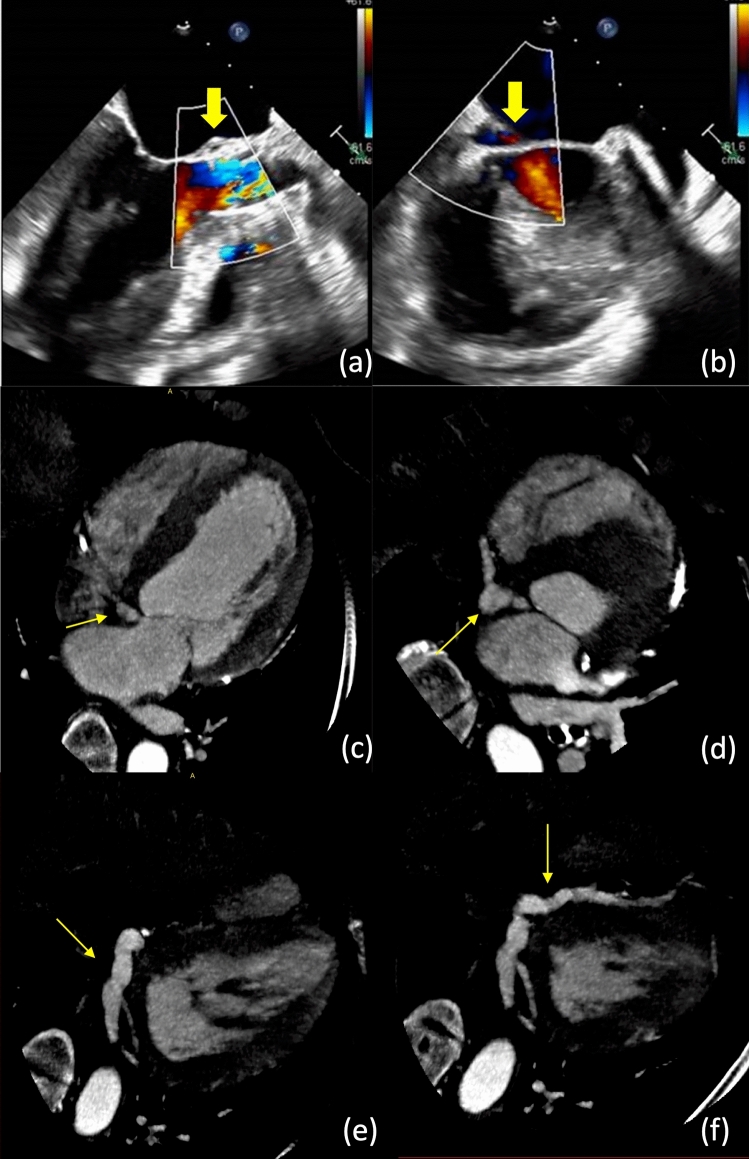
**a** Left ventricular and aortic valve transesophageal echocardiogram (TEE) view showing area of echolucency (yellow arrow) in the posterior aortic annulus area, bulging in systole (3-4 mm) in the right fibrous trigone. **b** In the same view as above, the echolucnecy is seen extending laterally and into the atrioventricular septum and lower atrial septum. **c−f** Serial cardiac computed tomography (CT) imaging (axial view) confirming the pseudoaneurysm (yellow arrow) communicates with the left ventricular outflow tract and travels posteriorly with connection to the left atrium, which then travels inferiorly with a pseudovascular distribution that occupies the space within the inferior interventricular groove

A 64-year-old woman with a past medical history of aortic stenosis status post (s/p) transcatheter aortic valve replacement (TAVR) (23 mm, Edwards SAPIEN 3), heart failure with reduced ejection fraction (25–30%), coronary artery disease with history of drug-eluting stents, end-stage-renal disease on hemodialysis, hypertension, hyperlipidemia, diabetes mellitus, and cigarette smoking of 40 years presented one year s/p TAVR and demonstrated clinical heart failure symptoms in hypertensive crisis. Blood cultures were positive for *Staphylococcus epidermidis* and transthoracic echocardiogram did not reveal any abnormalities including interventricular or native valve defects. However, transesophageal echocardiogram revealed areas of echolucency (Videos 1 and 2) that raised suspicion for aneurysm involving the mitral-aortic intervalvular fibrosa (MAIVF) (Figs. 1a, b). The bulging in systole (Video 3) implicated communication with the aortic system in the right fibrous trigone which raised suspicion for communication with the arterial system thus receiving arterial blood supply. Further workup with cardiac computed tomography revealed a fistula originating from the LV outflow tract that traveled posteriorly and inferiorly, forming a pseudoaneurysm in the planes of least resistance along the atrioventricular and interventricular grooves in an “arboreal” fashion, mimicking a vessel (Fig. 1c–f). The patient was managed conservatively with IV antibiotics for 4–6 weeks as surgery carried a prohibitive risk of cardiovascular mortality.

Infective endocarditis in patients with TAVR can result in abscess and fistula formation, which occur in 47% and 9% of TAVR patients, respectively [1]. Prior studies have demonstrated fistula formation between the aortic annulus to right atrium, and aortic annulus to left atrium; however, none have shown the unique arboreal-appearing, pseudovascular distribution as observed in this case [1–3].

We suspect several factors that promoted the arboreal distribution of the pseudoaneurysm simultaneously decreased the likelihood of rupture. First, the pseudoaneurysm after forming a connection with the left atrium, occupied space within the inferior interventricular groove, a path of least resistance. Next, as guided by Poiseuille’s Equation, the extensive length in arboreal morphology offset likelihood of rupture [4]. Last, the restrictive fistula bottleneck created a drop in pressure. A combination of above factors resulted in low pressures across the pseudoaneurysm and likely prevented catastrophic expansion and rupture. Our unique case may help raise a higher index of suspicion for pseudoaneurysms and close examination of the MAIVF in suspected aortic endocarditis in the setting of atypical clinical presentations, emphasizing the significance of understanding the physiological basis for morphological formations.

## Supplementary Information

Below is the link to the electronic supplementary material.

Supplementary file1 (DOCX 12 KB)

Supplementary file2 (MP4 1074 KB)

Supplementary file3 (MP4 908 KB)

Supplementary file4 (MP4 1130 KB)

## References

[1] Amat-Santos IJ, Ribeiro HB, Urena M (2015). Prosthetic valve endocarditis after transcatheter valve replacement: a systematic review. JACC Cardiovasc Interv.

[2] Mylotte D, Andalib A, Thériault-Lauzier P (2015). Transcatheter heart valve failure review: a systematic review. Eur Heart J.

[3] Chourdakis E, Koniari I, Hahalis G (2018). Endocarditis after transcatheter aortic valve implantation: a current assessment. J Geriatr Cardiol.

[4] Sutera SP, Skalak R (1993). The history of Poiseuille’s law. Annu Rev Fluid Mech.

