# Supplementary material for: An “arboreal” infective pseudoaneurysm following TAVR with “pseudovascular” distribution and morphology
Source: J Echocardiogr. 2022 Apr 6;21(2):94–6. doi: 10.1007/s12574-022-00571-y (PMC10195704; doi:10.1007/s12574-022-00571-y)
Supplement: Supplementary file 1 — Supplementary file1 (DOCX 12 KB) [file 12574_2022_571_MOESM1_ESM.docx]

**Video Title and Legends**

**Video 1:** 4 Chamber View

Four chamber view showing an area of echolucency along the anterior portion of the mitral valve annulus.

**Video 2:** LVOT with Color

Left ventricular outflow track view showing an area of echolucency within the mitral-aortic intervalvular fibrosa with Doppler color flow present during systole, indicating it's communication with the arterial blood supply and with LV as it fills during systole

**Video 3:** 3D Bulging During Systole

3D image showing a two small pockets with outward bulging occurring during systole contained within the mitral-aortic intervalvular fibrosa.
